# Risk Prediction Models and Novel Prognostic Factors for Heart Failure with Preserved Ejection Fraction: A Systematic and Comprehensive Review

**DOI:** 10.2174/1381612829666230830105740

**Published:** 2023-09-25

**Authors:** Shanshan Lin, Zhihua Yang, Yangxi Liu, Yingfei Bi, Yu Liu, Zeyu Zhang, Xuan Zhang, Zhuangzhuang Jia, Xianliang Wang, Jingyuan Mao

**Affiliations:** 1 Department of Cardiovascular, First Teaching Hospital of Tianjin University of Traditional Chinese Medicine/National Clinical Research Center for Chinese Medicine Acupuncture and Moxibustion, No. 88, Changling Road, Xiqing District, Tianjin 300381, China;; 2 Tianjin University of Traditional Chinese Medicine, No. 10, Poyang Lake Road, West Tuanpo New Town, Jinghai District, Tianjin 301617, China

**Keywords:** Heart failure with preserved ejection fraction, risk prediction model, prognostic factor, risk stratification, dynamic assessment, biomarkers

## Abstract

**Background:**

Patients with heart failure with preserved ejection fraction (HFpEF) have large individual differences, unclear risk stratification, and imperfect treatment plans. Risk prediction models are helpful for the dynamic assessment of patients' prognostic risk and early intensive therapy of high-risk patients. The purpose of this study is to systematically summarize the existing risk prediction models and novel prognostic factors for HFpEF, to provide a reference for the construction of convenient and efficient HFpEF risk prediction models.

**Methods:**

Studies on risk prediction models and prognostic factors for HFpEF were systematically searched in relevant databases including PubMed and Embase. The retrieval time was from inception to February 1, 2023. The Quality in Prognosis Studies (QUIPS) tool was used to assess the risk of bias in included studies. The predictive value of risk prediction models for end outcomes was evaluated by sensitivity, specificity, the area under the curve, C-statistic, C-index, *etc*. In the literature screening process, potential novel prognostic factors with high value were explored.

**Results:**

A total of 21 eligible HFpEF risk prediction models and 22 relevant studies were included. Except for 2 studies with a high risk of bias and 2 studies with a moderate risk of bias, other studies that proposed risk prediction models had a low risk of bias overall. Potential novel prognostic factors for HFpEF were classified and described in terms of demographic characteristics (age, sex, and race), lifestyle (physical activity, body mass index, weight change, and smoking history), laboratory tests (biomarkers), physical inspection (blood pressure, electrocardiogram, imaging examination), and comorbidities.

**Conclusion:**

It is of great significance to explore the potential novel prognostic factors of HFpEF and build a more convenient and efficient risk prediction model for improving the overall prognosis of patients. This review can provide a substantial reference for further research.

## 1 INTRODUCTION

Heart failure (HF), as the terminal stage of various cardiovascular diseases, portends a poor prognosis. Current data suggest that the prevalence of HF has been increasing due to an aging population and improved survival for diseases that can further develop into HF. This is followed by a sharp increase in the demand for medical services in the future, which will bring a heavy medical economic burden to the world. Epidemiological investigations showed different trends in HF incidence in patients with heart failure with reduced ejection fraction (HFrEF) (decreasing incidence) and heart failure with preserved ejection fraction (HFpEF) (increasing incidence), suggesting that HF requires more urgent attention.

HFpEF is considered to be a clinically heterogeneous syndrome associated with complex conditions, substantial utilization of healthcare resources, and premature mortality [1]. Data from a real-world study found that HFpEF has become an important subtype of HF worldwide in recent years, accounting for approximately 46% of heart failure hospitalization (HFH) [2]. Multimorbidity is common in both types of HF but is slightly more severe in HFpEF than in HFrEF. Correspondingly, the causes of death in HFpEF patients are more complex, and the proportion of non-cardiovascular diseases is higher than that in HFrEF patients [3]. Until now, HFpEF has lacked evidence-based treatment options and has a 50% mortality rate within five years of diagnosis. Therefore, optimal HFpHF prevention and control strategies should include quantification of risk in the individual patient, and early identification of high-risk patients is one of the potentially effective ways to improve prognosis.

Its incompletely understood pathophysiology has limited the accuracy of the diagnosis and the effectiveness of interventions. A comprehensive exploration of high-value prognostic factors is the first step in risk stratification and early intervention, which will facilitate the construction of a more complete risk-stratified evaluation system for HFpEF, to predict long-term outcomes and identify patients requiring strict clinical monitoring, promote a more personalized treatment, management and follow-up, dynamically adjust new therapeutic targets, and prolong survival and optimize the quality of life. To better capture future research hotspots, it may be worthwhile to review earlier work in this area. This study summarized the existing HFpEF risk prediction models for predicting long-term outcomes such as cardiovascular events, HFH, and all-cause mortality, and further reviewed the potential novel prognostic factors that could be used to optimize the existing models, providing a reference for related research of constructing a new model.

## 2 METHODS

The review was performed in accordance with the Preferred Reporting Items for Systematic Review and Meta-Analysis (PRISMA) extension statement (Hutton *et al.*, 2015). See Supplementary File for a completed PRISMA checklist.

### 2.1 Eligibility Criteria

a) The type of study was an original clinical trial, whether prospective or retrospective, to construct a new risk predictive model or to evaluate the performance (*e.g*. sensitivity, specificity, the area under the curve (AUC), C-statistic, C-index, *etc*.) of an existing model or to explore novel prognostic factors. b) The population was HFpEF patients in the stable phase or acute exacerbation phase. Gender, age, race, and hospitalization were not limited. c) Long-term end events such as deaths from various causes, hospitalizations from various causes, re-hospitalizations from various causes, cardiovascular and cerebrovascular events, *etc*. were observed.

### 2.2 Search Strategy

Studies on risk prediction models and prognostic factors for HFpEF were systematically searched. Relevant databases included PubMed and Embase. The retrieval time was from inception to February 1, 2023. The search strategy was developed according to the Cochrane Handbook for Systematic Reviews [4] by two researchers (Lin SS and Wang X). Search terms included heart failure, preserved ejection fraction, prognosis, prediction, and their synonyms. The synonyms in the group were connected by “or”, and the search terms between the groups were connected by “and”. Besides, the reference lists of related documents were tracked to avoid omission. Language is limited to English. The detailed search strategy is shown in Supplementary Material Tables **S1** & **S2**.

### 2.3 Literature Screening and Data Extraction

Records from databases will be managed by NoteExpress (V3.2.0). First, we excluded duplicate records. Second, by reading the title and abstract of each record, we excluded records that did not meet the eligibility criteria. Finally, we will download and read the full texts of potentially relevant studies to perform the second screening. The data from the eligible literature was extracted and recorded in a data extraction form developed in Microsoft Excel. The data extraction items included a title, first author, publication year, diagnostic criteria, sample size, age, gender, comorbidity, treatment, duration of follow-up, outcomes, model name, predictor variables, scoring method, performance, risk of bias information, and others. Literature screening and data extraction were done independently and cross-checked by two researchers (Lin SS and Wang X). Disagreement was determined through a discussion between the two investigators. When consensus cannot be reached, a third investigator assisted in the judgment. In the early stage of the study, we trained the evaluators and conducted pre-tests to ensure a standardized screening process.

### 2.4 Risk of Bias Assessment of Included Studies

The Quality in Prognosis Studies (QUIPS) tool [5] was used to assess the risk of bias of included studies from the following domains: study participation, study attrition, prognostic factor measurement, outcome measurement, study confounding, statistical analysis, and overall assessment. The results of the risk of bias assessment included the low, unclear, and high risk of bias. Risk of bias assessment was independently completed and cross-checked by two researchers (Lin SS and Wang X). Disagreements were resolved with the assistance of other researchers.

### 2.5 Exploration of Potential Novel Prognostic Factors

This review summarized the prognostic factors involved in existing models, explored potential novel prognostic factors with high value in the literature screening process, and classified and described them from several aspects, including demographic characteristics, lifestyle, laboratory tests, physical inspection, and comorbidities.

## 3 RESULTS

### 3.1 Basic Characteristics of Existing HFpEF Risk Prediction Models

**Fig. (1):**
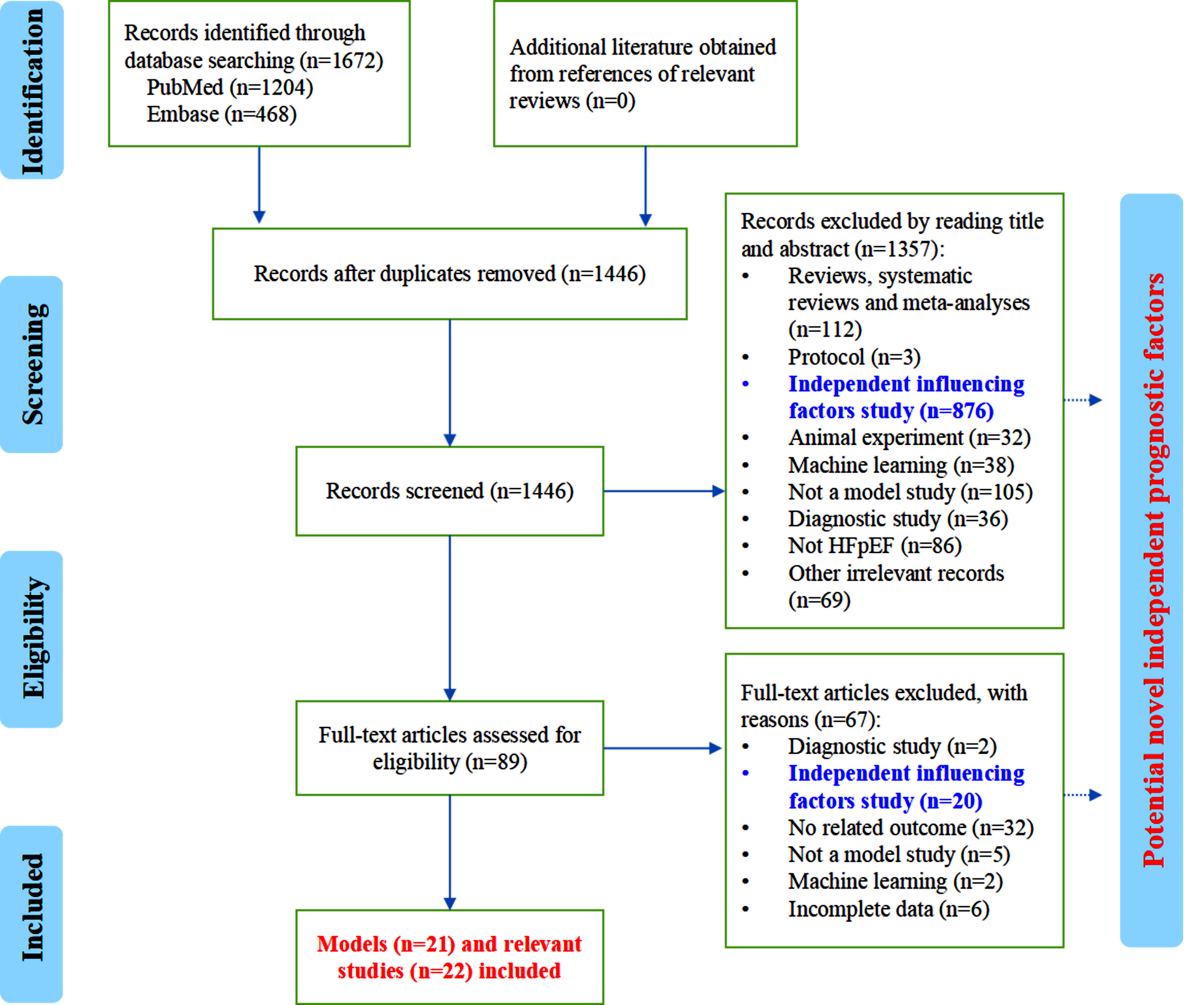
PRISMA flowchart of the literature screening process.

A total of 1,672 records were initially obtained. After reading the full text, 21 eligible HFpEF risk prediction models and 22 relevant studies were included in this review [6, 8-30] (Fig. **1**). Existing HFpEF risk prediction models included modified EFFECT score, MAGGIC risk score, a combination of MAGGIC risk score and P-selectin, H2FPEF score 1, H2FPEF score 2, 3A3B score, geriatric nutritional risk index (GNRI), controlling nutritional status score (CONUT) score + PF4, a combination of modified Glasgow prognostic score and NT-proBNP, SCD score, HAD-AFIB score, EPYC score, MEDIA echo score, HFA-PEFF score, a combination of HFA-PEFF score and PF3, DEI score model, CMR score, Essen stroke risk score (ESRS), and 3 undefined models. The follow-up period ranged from 6 months to 5.7 years (median follow-up time). The long-term outcomes included all-cause mortality, cardiovascular death, sudden cardiac death, pump failure death, heart failure hospitalization/rehospitalization, cardiovascular hospitalization, cardiovascular and cerebrovascular events, and so on. The predictive value was evaluated by sensitivity, specificity, the area under the curve (AUC), C-statistic, C-index, *etc*. Details are presented in Table **1**.

### 3.2 Results of Risk of Bias Assessment

In addition to the high risk of bias in the studies of Ghafoor [19], Liu [28] and the moderate risk of bias in the studies of Thorvaldsen [6], Kanagala [9], the overall risk of bias is considered as low for other studies proposing risk prediction models, as depicted in Table **2**.

### 3.3 Summary of Potential Novel Prognostic Factors for Long-term Outcomes of HFpEF

**Fig. (2):**
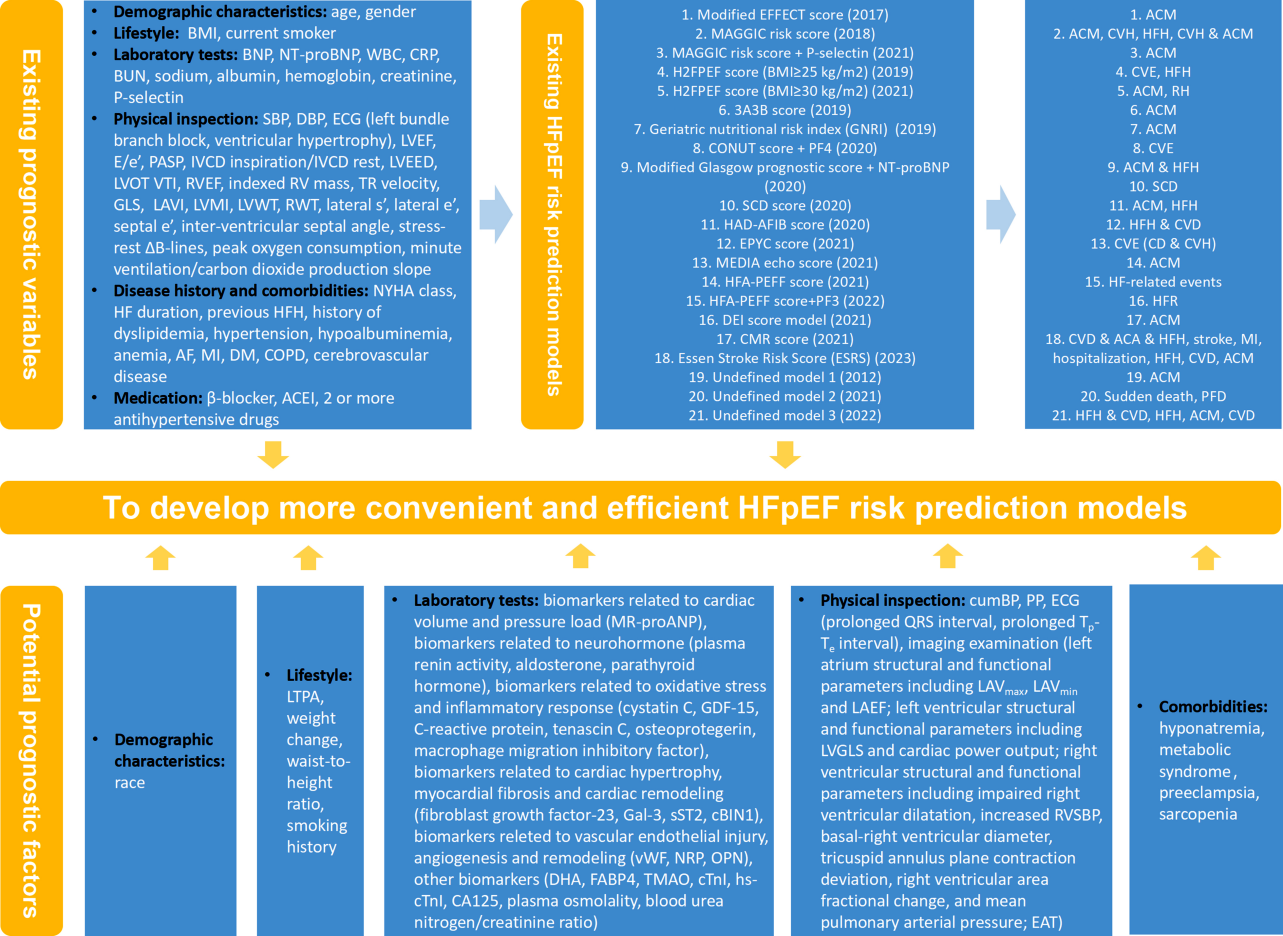
Combination of existing HFpEF risk prediction models and potential novel prognostic factors.**Abbreviations**: HFpEF, heart failure with preserved ejection fraction; BMI, body mass index; BNP, B-type natriuretic peptide; NT-proBNP, N-terminal pro B-type natriuretic peptide; WBC, white blood cell; CRP, C-reactive protein; BUN, blood urea nitrogen; SBP, systolic blood pressure; DBP, diastolic blood pressure; ECG, electrocardiogram; LVEF, left ventricular ejection fraction; E/e’, the ratio of peak mitral valve blood flow velocity in early diastole to peak mitral valve annulus velocity in early diastole; PASP, pulmonary artery systolic pressure; IVCD, inferior vena cava diameter; LVEED, left ventricle end-diastolic diameter; LVOT VTI, left ventricle outflow tract velocity-time integral; RV, right ventricular; RVEF, right ventricular ejection fraction; TR, tricuspid regurgitation; GLS, global longitudinal strain; LAVI, left atrial volume index; LVMI, left ventricular mass index; LVWT, left ventricular wall thickness; RWT, relative wall thickness; NYHA, New York Heart Association; HF, heart failure; AF, atrial fibrillation; MI, myocardial infarction; DM, diabetes mellitus; COPD, chronic obstructive pulmonary disease; ACEI, angiotensin-converting enzyme inhibitor; HFH, heart failure hospitalization; CVH, cardiovascular hospitalization; ACM, all-cause mortality; CCE, cardiovascular and cerebrovascular event; RH, rehospitalization; CVE, cardiovascular events; SCD, sudden cardiac death; CVD, cardiovascular death; CD, cardiac death; HFR, heart failure rehospitalization; PFD, pump failure death; ACA, aborted cardiac arrest; LTPA, leisure-time physical activity; GDF-15, growth differentiation factor-15; Gal-3, galectin 3; sST2, soluble suppression of tumorigenesis-2; cBIN1, cardiac bridging integrator 1; vWF, von Willebrand factor; NRP, neuropilin; OPN, osteopontin; DHA, docosahexaenoic acid; FABP4, fatty acid-binding protein 4; TMAO, trimethylamine N-oxide; cTnI, cardiac troponin I; hs-cTnI, high-sensitivity cardiac troponin I; CA125, carbohydrate antigen 125; cumBP, time-averaged cumulative blood pressure; PP, pulse pressure; T_p_-T_e_ interval, T wave peak-T wave end interval; LAV_max_, maximum left atrial volume; LAV_min_, minimum left atrial volume; LVGLS, left ventricular global longitudinal strain; RVSBP, right ventricular systolic blood pressure; EAT, epicardial adipose tissue.

In addition to risk predictive models, some novel clinical parameters with predictive value have also been discovered, which can be combined with existing models to obtain new models with higher predictive values in future research (Fig. **2**).

#### 3.3.1 Demographic Characteristics

Existing predictive models generally believe that advanced age and male gender are risk factors for adverse end-point events. However, it is worth noting that a study reported in the journal Circulation in 2018 retrospectively analyzed 12,417 HF patients from a prospective cohort study who were followed up for 11.6 years (median follow-up time) and found that there is no gender difference in the lifetime risk of HFpEF patients [31], which shows that the prognostic value of gender in HFpEF is uncertain. In addition, this clinical study also found that the lifetime risk of non-blacks in patients with HFpEF was approximately 1.5 times that of blacks, whereas this racial difference was not observed in patients with HFrEF [31].

#### 3.3.2 Lifestyle

##### 3.3.2.1 Leisure-time Physical Activity (LTPA)

Some scholars retrospectively analyzed the clinical data of 51,451 HF patients from 3 cohort studies and found that there is a strong dose-response relationship between the amount of LTPA and the risk of prognosis (HFR, cardiovascular death, and all-cause death) in patients with HFpEF. At least twice the minimum guideline-recommended amount of LTPA per week was associated with a 19% lower risk of poor prognosis compared with HFpEF patients who never performed LTPA (HR = 0.81, 95% confidence interval (CI): 0.68-0.97). In linear contrast analysis comparing the HRs associated with different LTPA levels for each HF subtype, a significant dose-dependent association was observed between LTPA levels and risk of HFpEF (p trend for HR = 0.006) but not HFrEF (p trend for HR = 0.182) [32]. In addition, the amount of LTPA in patients with HFpEF was negatively correlated with the risk of atrial fibrillation (AF). For every 10-fold increase in metabolic equivalent, the risk of AF may be reduced by 42.8% (HR = 0.572, 95% CI: 0.357-0.916, *P* = 0.020) [33].

##### 3.3.2.2 Body-size-related Indicators

Haass *et al.* retrospectively analyzed baseline data from 4109 patients with HFpEF in the I-PRESERVE study and found that the incidence of body mass index (BMI) and the composite endpoint (all-cause death and cardiovascular hospitalization) was a U-shaped relationship: when BMI was between 26.5 and 30.9 kg/m^2^, the incidence of composite endpoints was the lowest; when BMI < 23.5 kg/m^2^ or BMI ≥ 35 kg/m^2^ gradually, the incidence of the composite endpoint of patients increased with the trend [34]. Pandey *et al.* also demonstrated a U-shaped relationship by analyzing 997 patients from the TOPCAT trial, however, it was concluded that patients with HFpEF had the lowest risk of poor prognosis when BMI approached 25 kg/m^2^ [35]. There was also an association between weight change and the risk of poor prognosis in patients with HFpEF. People who lost ≥ 5% body weight (n = 241) within 1 year had a significantly higher risk of all-cause mortality compared with people with relatively stable body weight (n = 981) (HR = 1.42, 95% CI: 1.06-1.89, *P* = 0.002), while there was no significant change in the risk of all-cause mortality between people who gained ≥ 5% body weight (n = 293) within 1 year and those with relatively stable weight (HR = 0.98, 95% CI: 0.68-1.42, *P* = 0.932) [36]. In addition, a higher waist-to-height ratio is an independent risk factor for all-cause death (adjusted HR = 1.91, 95% CI: 1.06-3.45, *P* = 0.032), cardiovascular death (adjusted HR = 2.58; 95% CI 1.01-6.67, *P* = 0.048), and HF rehospitalization (adjusted HR = 3.04; 95% CI 1.26-7.31, *P* = 0.013) in HFpEF patients [37].

##### 3.3.2.3 Smoking History

Smoking is a poor prognostic factor for cardiovascular disease, and clinical data from 1717 HFpEF patients from the TOPCAT trial showed that patients with a smoking history had a significantly increased risk of poor prognosis compared with patients without a smoking history (n = 729). The risk of all-cause death, cardiac death, and HFR among people who have not quit smoking (n = 116) was about 81%, 76%, and 54% higher than those who had quit smoking (n = 871), respectively (HR = 1.81, 95% CI: 1.19-2.75; HR = 1.76, 95% CI: 1.04-2.98; HR = 1.54, 95% CI: 1.01-2.36) [38].

#### 3.3.3 Laboratory Tests

##### 3.3.3.1 Biomarkers Related to Cardiac Volume and Pressure Load

The natriuretic peptide family is a sensitive marker of cardiac volume load and pressure load, and family members include B-type natriuretic peptide (BNP) and N-terminal pro-B-type natriuretic peptide (NT-proBNP) is the most commonly used indicator to assess the prognosis of HF and has been used in HFpEF risk prediction models. Midregional pro-atrial natriuretic peptide (MR-proANP) is also a member of the natriuretic peptide family and has good stability as an intermediate product after the degradation of the N-terminal pro-A-type natriuretic peptide. Previous studies have shown that MR-proANP has potential applications in HF risk stratification and prognosis assessment [39]. Jensen *et al.* prospectively recruited 806 outpatients with type 2 diabetes mellitus and found that after 4.8 years of follow-up, patients with high MR-proANP (≥ 60 pmol/L) HFpEF had a significantly increased risk of cardiovascular events compared with non-HF patients (HR = 2.56, 95% CI: 1.64-4.00), while there was no significant change in the risk of cardiovascular events in patients with low MR-proANP (< 60 pmol/L) (HR = 2.18, 95% CI: 0.78-6.14) [40]. A small clinical trial prospectively enrolled 43 HFpEF patients to preliminarily explore a dose-effect relationship between MR-proANP and the incidence of a composite endpoint (HFR and all-cause death) in HFpEF patients. The results showed that when the MR-proANP level increased by 100 pmol/ml after adjusting for confounding factors, the incidence of composite endpoints increased by 58% (HR = 1.58, 95% CI: 1.02-2.28, *P* = 0.042) [41].

#### 3.3.3.2 Biomarkers Related to Neurohormone

Abnormal activation of the renin-angiotensin-aldosterone system (RAAS) is an important mechanism for the occurrence and development of HF. High plasma renin activity was found to be an independent predictor of all-cause death in HFpEF patients, regardless of RAAS inhibitor treatment (HR = 2.14, 95% CI: 1.20-3.82, *P* = 0.010). High plasma renin activity may cause overactivation of RAAS and continuous negative effects on the cardiovascular and renal systems, leading to poor outcomes in HFpEF patients [42]. The abnormal increase of its final product, aldosterone, can cause water and sodium retention and increase blood pressure, increase cardiac preload, and further lead to left ventricular remodeling and dysfunction [43]. A registry study of 873 patients showed that elevated aldosterone levels were associated with an increased risk of cardiac concentric remodeling in patients with HFpEF (odds ratio = 1.45, 95% CI: 1.03-2.04, *P* = 0.034) and were therefore considered to be an independent risk factor for all-cause death and the composite endpoint (all-cause death and HFR) (HR = 1.55, 95% CI: 1.06-2.27, *P* = 0.024; HR = 1.43, 95% CI: 1.11-1.85, *P* = 0.006) [44]. However, there is no reliable evidence that antagonists of the renin-angiotensin-aldosterone system improve long-term outcomes in patients with HFpEF.

The parathyroid hormone is also an important regulator of the functional state of the body. Altay *et al.* prospectively included 48 patients with confirmed HFpEF and found that in the multivariate logistic regression model, parathyroid hormone level was found to be an independent predictor of HFpEF (odds ratio = 1.300, *P* = 0.001). According to the ROC curve analyses: the optimal cut-off value of parathyroid hormone to predict HFpEF was found as > 68.4 pg/ml, with 91.7% sensitivity and 96.2% specificity (AUC = 0.979, 95% confidence interval = 0.928-0.997) [45].

##### 3.3.3.3 Biomarkers Related to Oxidative Stress and Inflamma-tory Response

Serum cystatin C is a member of the endogenous cystatin protease inhibitor family, which can participate in oxidative stress and inflammatory response and affect the process of HF. A study retrospectively analyzed 220 hospitalized patients with HFpEF. According to serum cystatin C levels, HFpEF patients were divided into two groups, namely the normal cystatin C group (serum cystatin C ≤ 1.15 mg/L) and cystatin C elevated group (serum cystatin C > 1.15 mg/L) and followed up for 180-420 days (median follow-up time 270 days). The study found that serum cystatin C level in HFpEF patients was significantly higher than that in healthy subjects. When the cut-off value was 1.20 mg/L, serum cystatin C showed the best predictive value in predicting all-cause mortality in HFpEF patients (AUC = 0.774, 95% CI: 0.667-0.882; sensitivity 70.59%, specificity 78.33%). Serum cystatin C combined with NT-proBNP can further improve the predictive ability of all-cause mortality in patients with HFpEF (AUC = 0.791, 95% CI: 0.683-0.900) [46].

Growth differentiation factor-15 is only slightly expressed in cardiac tissue under physiological conditions, and its expression is increased in cardiomyocytes and vascular tissue under pathological and environmental stress. It can regulate the process of inflammatory damage repair and the proliferation, differentiation, and remodeling of blood vessels through anti-oxidative stress and inflammatory responses [47, 48]. Studies have shown that increased growth differentiation factor-15 level is an independent risk factor for cardiovascular events, all-cause death, HF readmission, and composite endpoints (all-cause death and first HFR) in patients with HFpEF, and its prognostic value may be better than NT-proBNP [49-51].

In addition to C-reactive protein [52], P-selectin [9], tenascin C [53], osteoprotegerin [54], and macrophage migration inhibitory factors [55] are also involved in the body’s oxidative stress and inflammatory response and are independent prognostic factors in patients with HFpEF.

##### 3.3.3.4 Biomarkers Related to Cardiac Hypertrophy, Myocardial Fibrosis, and Cardiac Remodeling

Fibroblast growth factor-23 is mainly secreted by osteocytes and osteoblasts and can play an important role in angiogenesis, cardiac hypertrophy, fibrosis, and cardiac remodeling. A clinical trial prospectively enrolled 143 patients with HFpEF and 31 controls of similar age and sex, and the results showed that elevated fibroblast growth factor-23 levels were associated with female gender, increased AF frequency, elevated NT-proBNP levels, decreased hemoglobin levels, renal insufficiency, increased left atrial volume, impaired right ventricular systolic function, and myocardial fibrosis, which can be used as an independent risk factor for the composite endpoint (all-cause death and first HFH) and all-cause mortality in patients with HFpEF (HR = 3.44, 95% CI: 2.01-5.90, *P* < 0.001; HR = 2.85, 95% CI: 1.26-6.44, *P* = 0.012) [56].

Galectin 3 (Gal-3) is released by activated cardiac macrophages and can be involved in key processes such as myocardial fibrosis and cardiac remodeling [57]. Beltrami *et al.* prospectively enrolled 98 patients with AHF and found that elevated Gal-3 levels were significantly associated with the severity of diastolic dysfunction and LV stiffness in patients with HFpEF. Multivariate Cox regression analysis showed that LogGal-3 was an independent risk factor for the 6-month composite endpoint (cardiac death and readmission risk) in patients with HFpEF (HR = 19.62, 95% CI: 2.39-60.89, *P* = 0.006) [58].

Soluble suppression of tumorigenesis-2 (sST2), a member of the interleukin-1 receptor family, is a marker of ventricular mechanical stress. The secretion of sST2 in serum is markedly increased when cardiomyocytes are injured, myocardial fibrosis, or ventricular remodeling [59, 60]. The prognostic value of sST2 in both acute and chronic HF has been proven. When the cut-off value of sST2 is 63.02 pg/ml, it showed the best predictive value in predicting all-cause mortality in HFpEF patients (sensitivity 58.82%, specificity 81.77%) [46]; and its predictive ability to assess all-cause mortality, HFR, or worsening renal function in HF patients is superior to NT-proBNP [61].

Cardiac bridging integrator 1 (cBIN1) is a membrane-scaffolding protein in cardiomyocytes that organizes the dyad containing microdomains at the transverse tubules that are responsible for the initiation and regulation of systolic and diastolic calcium transients [62]. Due to the homeostatic release of extracellular cBIN1-microvesicles, the amount of cardiac t-tubule cBIN1-microdomains correlates to the level of cBIN1-vesicles in circulation. As a result, plasma cBIN1 levels are direct indicators of the number of myocardial t-tubule cBIN1-microdomains, reflecting a stable functional reserve of cardiomyocytes. Thus, its blood availability makes it an attractive biomarker for cardiomyocyte remodeling. In animal models of heart failure, as well as in biopsy specimens from patients with end-stage cardiomyopathy, CBIN1 levels are reduced [63, 64]. The association between the cBIN1 score, a dimensionless index based on the normalized reciprocal of cBIN1 plasma levels, and 1-year hospitalization rate in 52 HFpEF outpatients was analyzed. The results showed that patients with a cBIN1 score equal to or greater than 1.80 had an HRR of 3.8 for cardiovascular hospitalization compared with patients with a cBIN1 score less than 1.80 (95% CI, 1.3-11.2; *P* = 0.02) [65].

##### 3.3.3.5 Biomarkers Related to Vascular Endothelial Injury, Angiogenesis, and Remodeling

Von Willebrand factor (vWF) is a glycoprotein synthesized and secreted by vascular endothelial cells and megakaryocytes and is considered to be a marker of endothelial cell injury and dysfunction. In the early stage of HF, endothelial cell function damage can promote a large amount of vWF release. The higher the vWF level, the more severe the cardiac function impairment [66]. Kleber *et al.* assessed vWF levels in 457 patients with HFpEF and observed all-cause mortality in 40% of patients at a median follow-up of 9.7 years. Univariate Cox regression analysis showed that vWF levels were significantly positively correlated with all-cause mortality (HR/1 SD = 1.45, 95% CI: 1.26-1.68, *P* < 0.001). After adjusting for age, sex, BMI, NT-proBNP, renal function, and common comorbidities, vWF remained an independent risk factor for all-cause mortality in HFpEF patients (HR/1 SD = 1.22, 95% CI: 1.05-1.42, *P* = 0.001). The prognostic value of vWF combined with NT-proBNP is superior to NT-proBNP alone [67].

Angiogenesis-specific marker neuropilin (NRP) is a multifunctional cell surface co-receptor family, expressed by endothelial cells, immune cells, and vascular smooth muscle cells, and is a regulator of numerous signaling pathways in the vascular system [68]. Osteopontin (OPN), a specific marker of vascular remodeling, is a secreted glycosylated protein involved in many biological processes such as extracellular matrix deposition, soft tissue calcification, tissue fibrosis, tumor growth and metastasis, inflammatory response, and immune regulation. Studies have shown that the OPN has an important regulatory role in the progression of cardiac hypertrophy and fibrosis [69, 70]. Based on an *in vitro* study, López *et al.* found that OPN excess in HF patients was associated with left ventricular stiffness, systolic dysfunction, increased lysine oxidase, and insoluble collagen. OPN can also up-regulate lysine oxidase in human fibroblasts, promote the formation of insoluble collagen in HF patients, and change the mechanical properties of the left ventricle, further proving that OPN is closely related to HF [71]. A retrospective clinical trial analyzed 96 patients with HFpEF and found that both NRP and OPN could predict the 18-month risk of all-cause death or HFR in patients with HFpEF, suggesting that the development of HFpEF may be closely related to angiogenesis and vascular remodeling [72].

##### 3.3.3.6 Other Biomarkers

Lipid metabolism plays an important role in cardiovascular disease. Docosahexaenoic acid (DHA) is a polyunsaturated fatty acid that can improve blood circulation and regulate lipid metabolism, and its content is negatively correlated with obesity [73, 74]. A retrospective analysis of 93 patients with acute decompensated HFpEF found that DHA was a protective factor for all-cause mortality in HFpEF (HR = 0.16, 95% CI: 0.06-0.44, *P* = 0.001), which suggested that measuring serum DHA levels can help identify high-risk HFpEF patients, and DHA supplementation may be a potential treatment for high-risk HFpEF [75]. Fatty acid-binding protein 4 (FABP4), as a lipid chaperone, can participate in many biological processes such as lipid metabolism, inflammatory response, cell growth, and differentiation. A prospective study recruiting 92 patients with HFpEF and 20 patients with coronary artery disease without HF showed that HFpEF patients with FABP4 levels ≥ 43.5 ng/ml had significantly lower event-free survival than FABP4 levels < 43.5 ng/ml of patients, suggesting that elevated FABP4 levels were associated with the risk of the composite endpoint (all-cause death and HFH) in patients with HFpEF (log-rank *P* = 0.003) [76].

Trimethylamine N-oxide (TMAO) is one of the important metabolites of intestinal flora, and its metabolic process is closely related to the occurrence of cardiovascular diseases. Elevated TMAO levels can accelerate the progression of HF by inducing oxidative stress and inflammatory responses, promoting myocardial fibrosis, and interfering with lipid metabolism and mitochondrial energy metabolism, *etc*. [77]. A retrospective study of 823 patients showed that TMAO was not associated with prognosis in patients with HFpEF. However, Salzano *et al.* analyzed clinical data from a prospective cohort study of 118 patients with HFpEF and found that when the cut-off value of TMAO serum concentration was 5 mmol/l, increased TMAO serum concentration was significantly associated with the composite endpoint event risk in patients with HFpEF, both at 18 and 60 months of follow-up (adjusted HR = 3.66, 95% CI: 1.03-13.04, *P* = 0.045; adjusted HR = 2.45, 95% CI: 1.12-5.35, *P* = 0.025) and combined BNP can be used to assess risk stratification in patients with HFpEF [78].

In addition to the above biomarkers, there are many commonly used clinical indicators such as cardiac troponin I [79], high-sensitivity cardiac troponin I [80], carbohydrate antigen 125 [81], plasma osmolality [82], blood urea nitrogen/creatinine ratio [83], *etc*., which has potential application value in the prognostic assessment of HFpEF.

#### 3.3.4 Physical Inspection

##### 3.3.4.1 Blood Pressure

Current risk prediction models suggest that both systolic blood pressure (SBP) and diastolic blood pressure (DBP) are inversely associated with the incidence of adverse endpoints in HFpEF. However, clinical data from 3310 patients with HFpEF showed a U-shaped relationship between SBP levels and the risk of all-cause mortality and the composite endpoint (HFR and all-cause death) in HFpEF patients: when SBP was ~123 mmHg, the risk of all-cause death is lowest; when the SBP is 120-129 mmHg or 130-139 mmHg, there is a similar intermediate risk of all-cause mortality; when the SBP is 110-119 mmHg or ≥ 140 mmHg, the risk of all-cause mortality is highest [84]. In addition, Fuchida *et al.* observed 206 patients with acute decompensated HFpEF and found that DBP ≤ 77 mmHg was an independent risk factor for HF readmission (HR = 2.229, 95% CI: 1.021-4.867, *P* = 0.044) [85]. Similarly, clinical data from 3,330 participants in the TOPCAT trial showed a U-shaped relationship between time-averaged cumulative blood pressure and all-cause death, cardiovascular death, and HF hospitalization among patients with HFpEF; when the SBP is 120-129 mmHg and DBP is 70-79 mmHg, the risk is lowest [86].

Pulse pressure (PP) reflects arterial stiffness and is a potential predictor of prognostic risk in HFpEF. A study that analyzed 4796 patients with HFpEF from the PARAGON-HF trial and grouped patients according to PP quartiles showed a J-shaped relationship between PP levels and the risk of the composite endpoint (HFH and cardiovascular death) in patients with HFpEF: patients with a PP value of 50-57 mmHg had the best prognosis, while those with a PP value of 69-138 mmHg had the worst prognosis, and the composite endpoint, HFH, and myocardial infarction risk of the latter were 1.39, 1.43, 1.54 and times those of the former, respectively (HR = 1.39, 95% CI: 1.14-1.69, P = 0.001; HR = 1.43, 95% CI: 1.15-1.79, *P* = 0.001; HR = 1.54, 95% CI: 1.06-2.23, *P* = 0.022) [87].

##### 3.3.4.2 Electrocardiogram

An electrocardiogram is a visual representation of cardiac electrical activity. A secondary analysis was conducted to explore the association between resting heart rate and adverse outcomes in 2705 HFpEF patients from the TOPCAT trial [88]. The resting heart rate was obtained from baseline electrocardiogram data. The results showed that an increased risk of hospitalization (HR = 1.03, 95% CI = 1.004-1.060), hospitalization for heart failure (HR = 1.10, 95% CI = 1.05-1.15), death (HR = 1.10, 95% CI = 1.06-1.16) and cardiovascular death (HR = 1.13, 95% CI = 1.07-1.19) for per 5-beats per minute increase in heart rate. When further analysis was limited to patients who did not report using beta-blockers, the magnitude of the association for each outcome (per 5-bpm increase) did not change substantially. A meta-analysis combining data from 14,054 patients came to a similar conclusion that high resting heart rate is a risk factor for adverse outcomes in patients with HFpEF. In addition, further subgroup analyses indicated that these positive relationships were significant in patients with sinus rhythm but not in those with atrial fibrillation [89]; HFpEF patients with sinus rhythm may benefit from heart-lowering drugs such as ivabradine [90].

A prolonged QRS interval on the electrocardiogram indicates a delayed ventricular depolarization process that, if not corrected in time, may lead to changes in cardiac structure and function, ultimately leading to HF [91]. A Swedish Heart Failure Registry of 25171 patients showed a significant difference in survival between the QRS ≥ 120 ms and QRS < 120 ms groups (1-year follow-up: 77% *vs*. 82%, *P* < 0.001; 5-year follow-up: 42% *vs*. 51%, *P* < 0.001), suggesting that QRS interval prolongation is an independent risk factor for all-cause mortality in HFpEF patients (HR = 1.12, 95% CI: 1.05-1.19, *P* < 0.05) [92].

The T wave peak-T wave end interval (T_p_-T_e_ interval) reflects the dispersion of repolarization across the ventricular wall. Prolonged T_p_-T_e_ interval indicates a widening of the vulnerable period, which is prone to cause malignant arrhythmia events such as ventricular tachycardia or ventricular fibrillation, and is also independently associated with an increased risk of major adverse cardiovascular events in patients with HFpEF [93, 94].

##### 3.3.4.3 Imaging Examination

###### 3.3.4.3.1 Left Atrium Structural and Functional Parameters

Maximum left atrial volume is a guideline-recommended key parameter for assessing left atrial diastolic function. However, one study analyzed 347 patients with HFpHF from the TOPCAT trial and found that the minimum left atrial volume may be more efficient in risk identification than the maximum left atrial volume. For every 15.4 ml/m^2^ increase in minimum left atrial volume, the risk of the composite endpoint (cardiovascular death, survival of cardiac death, and HFH) and HFH increased by 35% and 42%, respectively (HR = 1.35, 95% CI: 1.12-1.61; HR = 1.42, 95% CI: 1.17-1.71). In addition, the left atrium emptying fraction was an independent protective factor for the composite endpoint in patients with HFpEF. For each 14.2%decrease in the left atrium emptying fraction, the risk of the composite endpoint and HFH increased by 63% and 104%, respectively [95].

###### 3.3.4.3.2 Left Ventricular Structural and Functional Parameters

The ratio of peak mitral valve blood flow velocity in early diastole to peak mitral valve annulus velocity in early diastole (E/e') value in echocardiography is a classic parameter for evaluating left ventricular diastolic function and has been used in the construction of risk prediction models for HFpEF. For left ventricular systolic dysfunction, abnormal left ventricular global longitudinal strain (LVGLS) can be used as an early marker. A secondary analysis of 206 HFpEF patients from a prospective study found that HFpEF patients with |LVGLS| < -8.5% had a higher incidence of the composite endpoint than patients with |LVGLS| ≥ -8.5%. And for every 1% increase in LVGLS, the risk of the composite endpoint increased by 1.05 times, which indicates that LVGLS is an independent risk factor for the composite endpoint (HFH and cardiovascular death) in patients with HFpEF (HR = 1.05, 95% CI: 1.00-1.10, *P* = 0.03) [96]. A reduced |LVGLS| is independently associated with the occurrence of clinical adverse outcomes (*P* < 0.001) [97, 98]. However, the optimal cut-off value remains to be explored. Cardiac power output is also a measure of left ventricular performance. Lower cardiac power output is independently associated with the composite of cardiovascular mortality or HF hospitalization (HR = 0.70 per 1 SD, 95% CI: 0.49-0.97, *P* = 0.03) [99].

###### 3.3.4.3.3 Right Ventricular Structural and Functional Parameters

Diastolic dysfunction caused by impaired right ventricular dilatation and increased right ventricular afterload is closely related to the development of HF. Harada *et al*. retrospectively recruited 322 patients with HFpEF and found that impaired right ventricular dilatation and increased right ventricular systolic blood pressure were independent risk factors for the composite endpoint (sudden death, death from HF, and HFH) (HR = 2.046, 95% CI: 1.237-3.385, *P* = 0.005; HR = 1.032/1 mmHg, 95% CI: 1.012-1.052, *P* = 0.002). Patients had the lowest event-free rate when right ventricular dilatation was low and right ventricular systolic blood pressure was elevated (≥ 35 mmHg) [100]. In a retrospective study analyzing 3-year follow-up data from 73 patients with acute HFpEF, multivariate Cox regression modeling found that increases in basal-right ventricular diameter and inferior vena cava diameter were significantly associated with all-cause mortality in patients with acute HFpEF (HR = 2.4, *P* = 0.04; HR = 1.06, *P* = 0.02) [101]. In addition, the results of the meta-analysis showed that the right ventricular function parameter tricuspid annulus plane contraction deviation (HR = 1.26/5 mm reduction, 95% CI: 1.16-1.38, *P* < 0.0001), right ventricular area fractional change (HR = 1.16/5% decrease, 95% CI: 1.08-1.24, *P* < 0.0001), mean pulmonary arterial pressure (HR = 1.26/5 mmHg increase, 95% CI: 1.15-1.38, *P* < 0.0001), and pulmonary artery systolic pressure (HR = 1.15/5% reduction, 95% CI: 1.12-1.18, *P* < 0.0001) were also independent risk factors for mortality in patients with HFpEF [102]. Measurement of these parameters can not only be used to assess right ventricular function but also help to adjust diuretic dosage in patients with HFpEF, relieve clinical symptoms, and improve hemodynamics in patients with right ventricular dysectasia.

###### 3.3.4.3.4 Epicardial Adipose Tissue (EAT)

EAT can regulate the physiological functions of coronary artery and myocardial tissue through paracrine and autocrine, and has potential value in the clinical treatment and prognosis evaluation of cardiovascular diseases [103]. EAT contains endogenous adrenergic and cholinergic nerves that interact with the extrinsic cardiac sympathetic and parasympathetic nervous systems [104]. Increased EAT thickness in HF is directly associated with cardiac sympathetic dysfunction [105]. In addition, the volume of EAT is also closely related to the severity of myocardial fibrosis [106]. Pugliese *et al.* used cardiopulmonary echocardiography combined with exercise stress to assess the EAT in HF patients with different ejection fractions and healthy subjects and found that the EAT thickness of HFpEF patients was higher than that of healthy subjects, while the EAT thickness of HFrEF patients was lower than that of healthy subjects. EAT thickness > 5 mm in patients with HFpEF was an independent risk factor for the composite endpoint (HFH and cardiovascular death) (HR = 1.12, 95% CI: 1.04-1.37) [107].

#### 3.3.5 Comorbidities

Common etiology and pathogenesis are the main reasons for the existence of comorbidities. Various comorbidities, such as severe cardiac insufficiency, anemia, AF, hypertension, diabetes mellitus, and chronic obstructive pulmonary disease, have been used as predictor variables in existing HFpEF risk prediction models. In addition, diseases such as hyponatremia [108], metabolic syndrome [109], preeclampsia [110], and sarcopenia [111] have also been validated to be associated with the prognosis of HFpEF.

## 4 DISCUSSION

With the in-depth exploration of the pathogenesis, pathological changes, and demographic characteristics of HFpEF, the development, and application of HFpEF prognostic risk models have become increasingly popular. These models come from three main sources:

1. Models from other research fields are directly applied to the risk prediction of HFpEF. For example, the “HFA-PEFF diagnostic algorithm” was originally a diagnostic tool for HFpEF [22], and some researchers found that it still has a certain clinical application value in the risk prediction of HFpEF; CONUT is a screening tool for controlling nutritional status [13], which can be used for daily automatic assessment of the nutritional status of hospitalized patients. By combining CONUT with other predictive variables, a new HFpEF risk prediction model was developed. However, the predictive value of such models may be largely limited by the substantial differences in the pathogenesis of different diseases.
2. By redefining the evaluation criteria or introducing new predictor variables, the prediction model is improved from the HFrEF model, which can combine the disease characteristics of HFpEF to achieve a more accurate prediction.
3. Prediction models developed specifically for HFpEF, which may require significant upfront investment to obtain sufficient clinical data to support them.

The research designs applied to HFpEF risk prediction model research mainly include the following:

1. Rating scales: By collecting clinical data, the researchers first screened the independent risk factors or protective factors of outcome indicators using statistical methods such as univariate or multivariate COX regression models, and then assigned different weights to them to generate the scoring method of this model. An alignment diagram is a special form of the rating scale, which is established based on multi-factor regression analysis, integrates multiple predictive indicators, and then draws the line segment with the scale on the same plane according to a certain proportion, to express the mutual relationship between various variables in the prediction model.
2. Machine learning: Since machine learning is a complex algorithm, models built through machine learning often require too many predictive variables, which limits their application in clinical practice, so this review does not summarize such models.

Although HFpEF risk prediction models are gradually increasing, they are not widely promoted and applied, which may be due to the following reasons:

1. There are too many predictive variables and the scoring method is too complicated; or the number of predictor variables is appropriate, but the predictive value is limited.
2. The predictive variables are unconventional inspection parameters, which are difficult to obtain. Given the above problems, researchers should be encouraged to explore predictive variables with high sensitivity and specificity. Even though these variables are difficult to obtain in clinical practice at present, they are still expected to be developed into accurate, reliable, and efficient routine clinical examinations with further research. It is helpful to develop a convenient and efficient risk prediction model to break through the limitations of existing clinical routine examination parameters.
3. Some prediction models have not been externally validated and their predictive value needs to be further confirmed.
4. The relevant information of the model is not comprehensive, the research process is not open and transparent, and only part of the results of many evaluation indexes related to the prediction efficiency is provided, which makes it difficult for other researchers to comprehensively evaluate its reliability and applicability. Given the above problems, researchers should be encouraged to provide comprehensive predictive capability evaluation results, including not only sensitivity and specificity but also comprehensive evaluation outcomes, such as accuracy, positive likelihood ratio, negative likelihood ratio, AUC, c-statistic, c-index, *etc*. In addition, researchers should provide evaluation results in both the derivation cohort and validation cohort.

A comprehensive exploration of high-value prognostic factors is the first step in risk stratification and early intervention, which will facilitate the construction of a more complete risk-stratified evaluation system for HFpEF that enables dynamic assessment of patients' prognostic risk and early intensive therapy in high-risk patients. A better insight into which factors relate to poor outcomes also could reveal more pathophysiology mechanisms and help refine phenotypes for targeting existing and potential novel treatment options. Moreover, early risk assessment at the time of hospital presentation may guide clinician, patient, and family decision-making and identify patients in need of more intensive monitoring and therapy or palliative interventions.

## CONCLUSION

It is of great significance to explore the potential novel prognostic factors of HFpEF and build a more convenient and efficient risk prediction model for improving the overall prognosis of patients, which deserves continuous attention.

## Figures and Tables

**Table 1:** Existing HFpEF risk prediction models.

**Model (Published Year)**	**Predictor Variables and ** **Scoring Method**	**Participant**	**Duration of Follow-up**	**Outcomes**	**Actual Results**	**Performance (95% CI)**			**References**
						**Sensitivity & Specificity**	**AUC/** **C-statistic**	**C-index for Model Discrimination**	
Modified EFFECT score (2017)	A web-based calculator is available at https://www.cscc.unc.edu/aric/.Age, SBP, BUN, sodium concentration, cerebrovascular disease, COPD, hemoglobin, heart rate, natriuretic peptide, underweight (defined as BMI < 18.5), hypoxia and white race.	Derivation cohort: 1852 from 2005-2011Validation cohort: 821 from 2012-2013	1 year	• 28-day ACM	Derivation cohort: 11%.Validation cohort: 8%.	NA	Derivation cohort: 0.76;Validation cohort: 0.73.	NA	[6]
	A web-based calculator is available at https://www.cscc.unc.edu/aric/.Age, SBP, BUN, sodium concentration, cerebrovascular disease, COPD, heart rate, natriuretic peptide, underweight (defined as BMI < 18.5), hypoxia, history of atrial fibrillation/flutter.	Derivation cohort: 1852 from 2005-2011Validation cohort: 821 from 2012-2013	1 year	1-year ACM	Derivation cohort: 34%.Validation cohort: 31%.	NA	Derivation cohort: 0.72;Validation cohort: 0.71	NA	
MAGGIC risk score (2018)	Age, male, BMI, SBP, LVEF, creatinine, current smoker, DM, COPD, NYHA class, HF duration > 18 months, β-blocker use, and ACEI use [7].	Validation cohort: 407 from 2008.3-2011.5	3.6 ± 1.8 years	• ACM	28% (115/407)	NA	NA	0.74 (0.68-0.80)	[8]
				CVH	43% (174/407)	NA	NA	0.66 (0.60-0.71)	
				HFH	32% (129/407)	NA	0.66	0.64 (0.58-0.69)	
				Composite endpoint of CVH & ACM	55% (224/406)	NA	0.65	0.72 (0.67-0.77)	
MAGGIC risk score + P-selectin (2021)	MAGGIC risk score + P-selectin (threshold value of > 35506 pg/ml)	Validation cohort: 130	1428 (1153-1663) days (median, IQR)	• ACM	During follow-up (median 1428 days), there were 38 deaths.	NA	MAGGIC scores: 0.647;P-selectin: 0.618;MAGGIC scores + P-selectin: 0.710.	NA	[9]
H2FPEF score 1 (2019)	Heavy (obesity, BMI ≥ 25 kg/m^2^), AF, age > 60 years, treatment with 2 or more antihypertensive drugs, E/e’ > 9 and echocardiographic PASP > 35 mmHg (AF: 3 points, obesity: 2 points, others: 1 each).Three groups: low- (0-3 points), intermediate- (4-6 points), and high-score (7-9 points) groups.	Validation cohort: 452	46.4 months (median)	• CVE	Low-score (0-3) group: 23 (13.9%);intermediate-score (4-6) group: 37 (21.6%);high-score (7-9) group: 23 (33.8%)	With a cut-off value of 5.5: 49.4% & 72.9%	0.625 (0.557-0.693)	NA	[10]
		-		HFH	Low-score (0-3) group: 10 (6.1%);intermediate-score (4-6) group: 24 (14.0%);high-score (7-9) group: 19 (27.9%)	With a cut-off value of 5.5: 58.5% & 72.4%	0.680 (0.600-0.759)	NA	
H2FPEF score 2 (2021)	Heavy (obesity, BMI > 30 kg/m^2^), AF, age > 60 years, treatment with 2 or more antihypertensive drugs, E/e’ > 9 and echocardiographic PASP > 35 mmHg (AF-3 points, obesity: 2 points, others: 1 each).Three groups: low- (0-1 points), intermediate- (2-5 points), and high-score (6-9 points) groups.	Validation cohort: 476 from 2015.1-2018.4	27.5 ± 11.3 months (mean ± SD)	ACM	Low-score (0-3) group: 3 (6.4%);intermediate-score (4-6) group: 29 (11.1%);high-score (7-9) group: 31 (18.6%)	With a cut-off value of 5.5: 68.3% & 55.4%	0.67 (0.60-0.73), *P <* 0.0001	NA	[11]
				RH	Low-score (0-3) group: 28 (59.6%);intermediate-score (4-6) group: 159 (60.7%);high-score (7-9) group: 124 (74.3%)	With a cut-off value of 5.5: 50.5% & 66.7%	0.59 (0.54-0.65), *P* = 0.001	NA	
3A3B score (2019)	Age ≥ 75 years (2 points); albumin < 3.7 g/dl, anemia, BMI < 22 kg/m^2^, BNP ≥ 300 pg/ml (or NT-proBNP ≥ 1400 pg/ml), and BUN ≥ 25 mg/dl (1 point for each).The scores 1, 2, 3, and 4 predict 15, 25, 35, and 45% ACM at 5 years, respectively. At 1, 3, and 5 years, patients with 0 points had predicted ACM of 0.5, 2.5, and 9.5% respectively, while those with 6-7 points had predicted ACM of 22.0, 49.0, and 79.2%, respectively.	Derivation cohort: 1277 from the CHART-2 study;Validation cohort: 835 from the TOPCAT trial and 170 from the ASIAN-HF registry	5.7 years (median)	• ACM	Derivation cohort: 576 deaths occurred.	NA	NA	Derivation cohort: 0.708;Validation cohorts: 0.652 in TOPCAT trial and 0.741 in ASIAN-HF registry.	[12]
Geriatric nutritional risk index (GNRI) (2019)	GNRI = 14.89 × serum albumin (g/dL) + 41.7 × BMI/22.HFpEF patients with low GNRI (< 92, n = 49) with moderate or major nutrition-related risk and patients with high GNRI (≥ 92, n = 61) with low or no nutrition-related risk.	Validation cohort: 110	503.5 (IQR 328.0-790.0) days	ACM	24 deaths occurred.	NA	GNRI: 0.75Adding the GNRI to the logBNP: 0.80 (*P* = 0.040, n = 105).	NA	[13]
Controlling nutritional status score (CONUT) score+PF4 (2020)	CONUT score: three biomarkers including protein metabolism, immunocompetence, and lipid metabolism [14].PF4: age, previous HFH, DM, and lnBNP.Normal group (the CONUT score 0-1 group with a normal nutritional state), light group (the CONUT score 2-4 group with a light degree of under nutrition), moderate group (the CONUT score 5-8 group with a moderate degree of under nutrition), high group (the CONUT score 9-12 group with a severe degree of under nutrition).	Validation cohort: 506	1500 days (median: 1159 days)	• CVE	238 CVEs occurred.	With a cut-off value of 2.5: 38.2% & 76.9%	CONUT score: 0.599 (0.550-0.648);PF4: 0.608 (0.559-0.658)PF4 + CONUT score ≥ 2.5: 0.643 (0.594-0.691)	NA	[15]
Modified Glasgow prognostic score + NT-proBNP (2020)	Both elevated CRP (> 1 mg/dl) and hypoalbuminemia (< 3.5 g/dl) (2 points); only CRP > 1 mg/dl (1 point); hypoalbuminemia (< 3.5 g/dl) (0 points).+ NT-proBNPmGPS was scored as 0, 1, or 2 based on CRP and albumin levels.	Validation cohort: 315	1 year	• Composite endpoint of ACM & HFH	42 (13.3%)	NA	0.822	0.785	[16]
SCD score (2020)	• The dichotomous variables were coded as 0 (no) or 1 (yes). SCD score = 0.025 (age) + 0.575 (male) + 0.521 (DM) + 0.425 (MI) + 0.488 (left bundle branch block) + 0.424 (lnNT-proBNP).• High risk (score ≥ 5.6) and low risk (score < 5.6).	Derivation cohort: 4128 from the I-PRESERVE trial	4.1 years	• 5-year SCD	• 837 (24%) participants were categorized as high-risk.• The 5-year cumulative incidences of SCD was 6.3% (11% in the higher risk group *vs.* 4% in the lower risk group).	NA	NA	0.75	[17]
		Validation cohort: 615 from the TOPCAT trial	2.9 ± 1.3 years (mean ± SD)	5-year SCD	216 (35.1%) participants were categorized as high-risk.The 5-year cumulative incidences of SCD was 7.3% (3.7% to 12.4%) (15.2% (95% CI: 6.6-27.2) in the higher risk group *vs.* 2.8% (95% CI: 1.3-6.5) in the lower risk group).	NA	NA	0.74	[18]
HAD-AFIB score (2020)	Hypertension, age, diastolic dysfunction, admission for HF, filtration rate, ischemic heart disease, BMI (Supplementary Material Table **S3**).	Derivation cohort: 803Validation cohort: 402	41 months (median)	• ACM, HFH	NA	NA	Validation cohort: 0.72 (0.67-0.78) for ACM; 0.77 (0.70-0.83) for HFH.	NA	[19]
EPYC score (2021)	Stress-rest ΔB-lines > 10 (3 points), peak oxygen consumption < 16 ml/kg/min (2 points), minute ventilation/carbon dioxide production slope ≥ 36 (2 points), peak PAP_S_ ≥ 50 mmHg (1 point), and resting NT-proBNP > 900 pg/ml (1 point).Three groups: low-risk (<3 points), intermediate-risk (3-6 points), and high-risk group (>6 points).	Derivation cohort: 274	18.5 months (median)	• Composite endpoint of HFH & CVD	71 HFH and 10 CVD were observed.The event-free survival probability for low risk (EPYC score < 3 points), intermediate risk (3-6 points), and high risk (> 6 points) were 93%, 52%, and 20%, respectively.	NA	0.92 (0.88-0.96)	NA	[20]
MEDIA echo score (2021)	Four echocardiographic parameters: PAP_S_ > 40 mmHg, IVCD inspiration/IVCD rest ≥ 0.5, average E/e’ > 9, and lateral mitral annular s’ < 7 cm/s (1 point for each). The MEDIA echo score ranges from 0 to 4 (each criterion scores 1 point).Patients with an echo score ≥ 3 had a risk of 37.8% (95% CI: 27.9-46.3%), whereas patients with a score of 0/1 had a risk of 7.9% (95% CI: 2.4-13.1%).	Derivation cohort: 515	361 days (median)	• 1-year CVE (CD & CVH)	CVH occurred in 82 patients (16.6%), among which 33 (6.7%) were HFH. 101 patients (20.9%) reached a primary endpoint.	NA	NA	0.703 (0.675-0.730)	[21]
HFA-PEFF score (2021)	The score has functional, morphological, and biomarker domains. Within each domain, a major criterion scores 2 points or a minor criterion 1 point *(Supplementary Material Table* ***S4****)* [22].Low (0-1 points), intermediate (2-5 points), and high (6-9 points) score groups.	Validation cohort: 358 from 2015.1-2018.4	26.9 months (mean)	• ACM	63 patients (13.2%) died (3 (6.4%), 29 (11.1%), and 31 (18.6%) for low, intermediate, and high score groups).	With a cut-off value of 3.5: 78.3% & 54.8%	0.726 (0.651-0.080, *P <* 0.0001)	NA	[23]
HFA-PEFF score+PF3 (2022)	HFA-PEFF score: *Supplementary Material Table* ***S4*** [18].PF3: age + diabetes mellitus + previous hospitalization for HF.Two groups: intermediate-score (2-4 points), and high-score (5-6 points) groups.	Validation cohort: 502 hospitalizations from 2007.1-2013.9	1500 days (median: 1159)	• HF-related events	311 (62.0%) and 191 (38.0%) participants were categorized as intermediate- and high-score groups.236 CVEs were recorded during the follow-up period (median, 1,157 days).	HFA-PEFF score with a cut-off value of 4.5: 57.8% and 67.4%.	HFA-PEFF score: 0.633 (0.574-0.692; *P <* 0.001)	PF3: 0.598 (0.539-0.657);HFA-PEFF score ≥ 5 + PF3: 0.630 (0.571-0.689; *P* = 0.021).	[24]
DEI score model (2021)	DEI score = −28.763 + 4.558 × log (LVEED (mm)) + 1.961 × (E/e’ ratio) + 1.759 × log (LVOT VTI (cm)).Log odds ratio (for HFR at 6 months) = −8.394 + 0.114 × E/e’ ratio + 0.088 × LVEDD + 0.087 × VTI LVOT.	Derivation cohort: 91;Validation cohort: 46.	6 months	• HFR	Derivation cohort: 30 (33%)	With a cut-off value of ≥ -0.747: 73.33% (54.10-87.70) & 72.13% (59.20-82.90)	Derivation cohort: 0.746 (0.640-0.853);Validation cohort: 0.690 (0.520-0.861)	NA	[25]
CMR score (2021)	RVEF ≤ 49%, indexed RV mass > 17 g/m^2^, inter-ventricular septal angle > 139 (1 point for each).	Derivation cohort: 116 from 2012.4-2017.4.	3 ± 2 years (mean)	• ACM	61 (53%)	With a cut-off value of > 2: 72% (59.2-82.9) & 71% (57.1-82.4)	0.76 (0.67-0.83, *P* < 0.001)	NA	[26]
Essen stroke risk score (ESRS) (2023)	Age (1 point for 65-75 years, and 2 points for >75 years), current smoking (1 point), and a history of hypertension, diabetes mellitus, peripheral artery disease, MI, previous TIA/ stroke, or other cardiovascular diseases except for MI and AF at baseline (1 point for each).	Validation cohort: 3441	3.3 years	• Composite outcome of CVD, aborted cardiac arrest, or HFH.	1-2 points: 48/523;3 points: 130/958;4 points: 211/1061;5 points: 170/606;≥ 6 points: 112/293	NA	NA	0.69	[27]
-	-	-	-	• Any stroke	1-2 points: 6/523;3 points: 25/958;4 points: 43/1061;5 points: 28/606;≥ 6 points: 15/293	NA	NA	0.68	-
				• MI	1-2 points: 3/774;3 points: 19/919;4 points: 40/911;5 points: 39/558;≥ 6 points: 28/279	NA	NA	0.75	
				• Any hospitalization	1-2 points: 159/523;3 points: 348/958;4 points: 507/1061;5 points: 343/606;≥ 6 points: 201/293	NA	NA	0.68	
				• HFH	1-2 points: 31/523;3 points: 75/958;4 points: 151/1061;5 points: 114/606;≥ 6 points: 80/293	NA	NA	0.71	
				• CVD	1-2 points: 27/523;3 points: 70/958;4 points: 98/1061;5 points: 81/606;≥6 points: 60/293	NA	NA	0.68	
				• ACM	1-2 points: 38/523;3 points: 96/958;4 points: 166/1061;5 points: 137/606;≥ 6 points: 92/293	NA	NA	0.68	
Undefined model 1 (2012)	Hypoalbuminemia (6 points), history of HF (3.5 points), history of cerebrovascular disease (3.5 points), blood urea nitrogen >10 mmol/L (3.5 points), not using calcium channel blockers (3.5 points), not using angiotensin-converting enzyme inhibitor or angiotensin II receptor blockers (2.5 points), age > 78 years (2 points).Three groups: low risk (0 to 7.5 points), intermediate risk (8 to 11.5 points), and high risk (12 to 24.5 points) groups.	Derivation cohort: 727 from 2001-2010Validation cohort: 311 from 2001-2010	1 year	• 1-year ACM	Derivation cohort: 11%, 18%, and 41% for low, intermediate, and high-risk groups.Validation cohort: 13%, 23%, and 38% for low, intermediate, and high-risk groups.	NA	Derivation cohort: 0.72;Validation cohort: 0.68.	NA	[28]
Undefined model 2 (2021)	• Risk score for sudden death = 0.034a + 0.506b + 0.036c + 0.568e + 0.463f + 0.34g + 0.048i.• Risk score for PFD = 0.044a + 0.028d + 0.758e + (-0.676)f + 0.06h + 0.059i.• Older age (age 60 years or above, per 1-year increase); (b) male; (c) LVEF 45-60%, per 1% decrease; (d) DBP up to 80 mmHg, per 1 mmHg decrease; (e) history of DM; (f) history of dyslipidemia; (g) left ventricular hypertrophy on electrocardiography; (h) serum creatinine 0.8-2.5 mg/dl, per 0.1 mg/dl increase; (i) NT-proBNP up to 3000 pg/ml, per 100 pg/ml.	Derivation cohort: 4116 from the I-PRESERVE trialValidation cohort: 3401 from TOPCAT trial	Derivation cohort: 52.9 months (median);Validation cohort: 41.1 months (median)	• Sudden death	Derivation cohort: 877 death events occurred, including 230 sudden deaths and 123 PFDs.Validation cohort: 520 death events occurred, including 110 sudden deaths and 65 PFDs.	NA	NA	Derivation cohort: 0.75 (0.72-0.78);Validation cohort: 0.73 (0.64-0.83)	[29]
				PFD		NA	NA	Derivation cohort: 0.80 (0.76-0.84);Validation cohort: 0.80 (0.68-0.92)	
Undefined model 3 (2022)	1 −[0.99932^exp (0.46 × log hs-cTnT+ 0.48 × log NT-proBNP+ 0.51 × NYHA class+ 0.54 × recent HFH1+ 0.67 × recent HFH2+ 0.53 × COPD + 0.48 × DM (insulin)+ 0.13 × DM (without insulin)+ 0.34 × HF diagnosis+ 0.35 × haemoglobin −0.29 × empagliflozin)].‘Recent HFH1’ and ‘recent HFH2’ are indicator variables for whether the most recent HHF was within 3-6 or <3 months.	Derivation cohort: 5988 in EMPEROR-Preserved trial from 2017.3-2020.4.Validation cohort: 1251 in PARAGON-HF trial from 2014.6-2016.12.	Derivation cohort: 26.2 months (median).Validation cohort: 24 months.	Composite outcome of HFH or CVD	Derivation cohort: 926 of 5988 patients (15.5%), 415 in the empagliflozin group, and 511 in the placebo group (HR 0.79, 0.69-0.90, *P <*0.001).Validation cohort: 223 (17.8%).	NA	Derivation cohort: 0.748 (95% CI 0.732-0.764).Validation cohort: 0.711 (0.672-0.749).	NA	[30]
-	1 - [0.99973^exp (0.57 × log hs-cTnT + 0.49 × log NT-proBNP + 0.66 × NYHA class + 0.80 × recent HHF1 + 1.02 × recent HHF2 + 0.58 × COPD + 0.44 × HF diagnosis + 0.48 × haemoglobin -0.39 × Empagliflozin)	Derivation cohort: 5988 in EMPEROR-Preserved trial from 2017.3-2020.4.Validation cohort: 1251 in PARAGON-HF trial from 2014.6-2016.12.	Derivation cohort: 26.2 months (median).Validation cohort: 24 months.	• HFH	NA	NA	Derivation cohort: 0.787 (95% CI 0.769,0.805).Validation cohort: 0.712 (0.668-0.757).	NA	-
-	1 −[0.99859^exp (0.42 × log hs-cTnT+ 0.34 × log NT-proBNP × 0.33 × NYHA class+ 0.54 × COPD + 0.07 × (4.5 −Albumin)/0.1)+ 0.18 ×Age1+ 0.45 ×Age2+ 0.31 × LVEF+ 0.35 × DM (insulin)+ 0.02 × DM (without insulin)], where ‘Age1’ and ‘Age2’ are indicator variables for whether the patient’s age is 65 to <75 or ≥75 years, respectively.	Derivation cohort: 5988 in EMPEROR-Preserved trial from 2017.3-2020.4.Validation cohort: 1251 in PARAGON-HF trial from 2014.6-2016.12.	Derivation cohort: 26.2 months (median).Validation cohort: 24 months.	• ACM	Derivation cohort: 849 of 5988 patients (14.2%), 422 in the empagliflozin group and 427 in the placebo group(HR 1.00, 0.87-1.15).Validation cohort: 143 (11.4%).	NA	Derivation cohort: 0.715 (95% CI 0.697, 0.733).Validation cohort: 0.719 (0.665-0.772).	NA	-
-	1 - [0.9994^exp (0.37 × log hs-cTnT + 0.40 × log NT-proBNP + 0.29 × NYHA class + 0.55 × COPD + 0.07 × ((4.5 - Albumin)/0.1) + 0.26 × Age1 + 0.36 × Age2 + 0.41 × LVEF + 0.36 × DM (insulin) - 0.09 × DM (without insulin)], where ‘Age1’ and ‘Age2’ are indicator variables for whether patient´s age is 65 to <75 or >=75 years old, respectively.	Derivation cohort: 5988 in EMPEROR-Preserved trial from 2017.3-2020.4.Validation cohort: 1251 in PARAGON-HF trial from 2014.6-2016.12.	Derivation cohort: 26.2 months (median).Validation cohort: 24 months.	• CVD	Derivation cohort: 463 of 5988 patients (7.7%), 219 in empagliflozin, and 244 in placebo (HR 0.91, 0.76-1.09).Validation cohort: 83 (6.6%).	NA	Derivation cohort: 0.718 (95% CI 0.694, 0.741).Validation cohort: 0.718 (0.652-0.784).	NA	-

**Abbreviations**: HFpEF, heart failure with preserved ejection fraction; ADHFpEF, acute decompensated heart failure with preserved ejection fraction; HFnEF, heart failure with normal ejection fraction; HFmrEF, heart failure with mid-range ejection fraction; SBP, systolic blood pressure; BUN, blood urea nitrogen; COPD, chronic obstructive pulmonary disease; BMI, body mass index; LVEF, left ventricular ejection fraction; DM, diabetes mellitus; NYHA, New York Heart Association; HF, heart failure; ACEI, angiotensin-converting enzyme inhibitor; AF, atrial fibrillation; E/e’, the ratio of peak mitral valve blood flow velocity in early diastole to peak mitral valve annulus velocity in early diastole; PASP, pulmonary artery systolic pressure; BNP, B-type natriuretic peptide; NT-proBNP, N-terminal pro B-type natriuretic peptide; CRP, C-reactive protein; PF4, 4 prognostic factors; MI, myocardial infarction; IVCD, inferior vena cava diameter; LVEED, left ventricle end-diastolic diameter; LBBB, left bundle branch block; LVOT VTI, left ventricle outflow tract velocity-time integral; RV, right ventricular; RVEF, right ventricular ejection fraction; DBP, diastolic blood pressure; WBC, white blood cell; HFH, heart failure hospitalization; CVH, cardiovascular hospitalization; ACM, all-cause mortality; CCE, cardiovascular and cerebrovascular events; RH, rehospitalization; CVE, cardiovascular events; SCD, sudden cardiac death; CVD, cardiovascular death; CD, cardiac death; HFR, heart failure rehospitalization; PFD, pump failure death; IQR, inter-quartile range; SD, standard deviation; AUC, areas under the curve; HR, hazard ratio; CI, confidence interval; NRI, net reclassification improvement; IDI, integrated discrimination improvement; ROC, receiver operating characteristic; C-index, index of concordance; & refers to composite endpoint; NA, not available.

**Table 2:** Risk of bias assessment for studies proposing risk prediction models.

**Study ID**	**Study ** **Participation**	**Study ** **Attrition**	**Prognostic Factor Measurement**	**Outcome ** **Measurement**	**Study ** **Confounding**	**Statistical ** **Analysis**
Thorvaldsen (2017) [6]	Low	Moderate	Low	Low	Low	Low
Rich (2018) [8]	Low	Low	Low	Low	Low	Low
Kanagala (2021) [9]	Moderate	Moderate	Low	Low	Low	Moderate
Sueta (2019) [10]	Low	Low	Low	Low	Low	Low
Sun (2021) [11]	Low	Low	Low	Low	Low	Low
Kasahara (2019) [12]	Low	Low	Low	Low	Low	Low
Nishi (2019) [13]	Low	Low	Low	Low	Low	Low
Komorita (2020) [15]	Low	Low	Low	Low	Low	Low
Bolat (2020) [16]	Low	Low	Low	Low	Low	Low
Adabag (2014) [17]	Low	Low	Low	Low	Low	Low
Adabag (2020) [18]	Low	Low	Low	Low	Low	Low
Ghafoor (2020) [19]	High	Moderate	Moderate	Low	Low	Moderate
Pugliese (2021) [20]	Low	Low	Low	Low	Low	Low
Huttin (2021) [21]	Low	Low	Low	Low	Low	Low
Sun (2021) [23]	Low	Low	Low	Low	Low	Low
Egashira (2022) [24]	Low	Low	Low	Low	Low	Low
Zamfirescu (2021) [25]	Low	Low	Low	Low	Low	Low
Garg (2021) [26]	Low	Low	Low	Low	Low	Low
Zhu (2023) [27]	Low	Low	Low	Low	Low	Low
Liu (2012) [28]	High	Moderate	Moderate	Low	Moderate	Moderate
Shen (2021) [29]	Low	Low	Low	Low	Low	Low
Pocock (2022) [30]	Low	Low	Low	Low	Low	Low

## LIST OF ABBREVIATIONS

EAT: Epicardial Adipose Tissue
HF: Heart Failure
HFpEF: Heart Failure with Preserved Ejection Fraction
QUIPS: Quality in Prognosis Studies

## SUPPLEMENTARY MATERIAL

PRISMA checklist is available as supplementary material on the publisher’s website along with the published article.

## References

[1] Shah S.J., Katz D.H., Deo R.C. (2014). Phenotypic spectrum of heart failure with preserved ejection fraction.. Heart Fail. Clin..

[2] Shah K.S., Xu H., Matsouaka R.A. (2017). Heart failure with preserved, borderline, and reduced ejection fraction: 5-year outcomes.. J. Am. Coll. Cardiol..

[3] Dunlay S.M., Roger V.L., Redfield M.M. (2017). Epidemiology of heart failure with preserved ejection fraction.. Nat. Rev. Cardiol..

[4] Higgins J., Thomas J. (2020). Cochrane Handbook for Systematic Reviews of Interventions.. Cochrane.

[5] Hayden J.A., van der Windt D.A., Cartwright J.L., Côté P., Bombardier C. (2013). Assessing bias in studies of prognostic factors.. Ann. Intern. Med..

[6] Thorvaldsen T., Claggett B.L., Shah A. (2017). Predicting risk in patients hospitalized for acute decompensated heart failure and preserved ejection fraction: The atherosclerosis risk in communities study heart failure community surveillance.. Circ. Heart Fail..

[7] Pocock S.J., Ariti C.A., McMurray J.J.V. (2013). Predicting survival in heart failure: A risk score based on 39 372 patients from 30 studies.. Eur. Heart J..

[8] Rich J.D., Burns J., Freed B.H., Maurer M.S., Burkhoff D., Shah S.J. (2018). Meta-Analysis Global Group in Chronic (MAGGIC) heart failure risk score: Validation of a simple tool for the prediction of morbidity and mortality in heart failure with preserved ejection fraction.. J. Am. Heart Assoc..

[9] Kanagala P., Arnold J.R., Khan J.N. (2021). Plasma P‐selectin is a predictor of mortality in heart failure with preserved ejection fraction.. ESC Heart Fail..

[10] Sueta D., Yamamoto E., Nishihara T. (2019). H2FPEF score as a prognostic value in HFpEF patients.. Am. J. Hypertens..

[11] Sun Y., Wang N., Li X. (2021). Predictive value of H2 FPEF score in patients with heart failure with preserved ejection fraction.. ESC Heart Fail..

[12] Kasahara S., Sakata Y., Nochioka K. (2019). The 3A3B score: The simple risk score for heart failure with preserved ejection fraction - A report from the CHART-2 Study.. Int. J. Cardiol..

[13] Nishi I., Seo Y., Hamada-Harimura Y. (2019). Geriatric nutritional risk index predicts all‐cause deaths in heart failure with preserved ejection fraction.. ESC Heart Fail..

[14] Ignacio de Ulíbarri J., González-Madroño A., de Villar N.G. (2005). CONUT: A tool for controlling nutritional status. First validation in a hospital population.. Nutr. Hosp..

[15] Komorita T., Yamamoto E., Sueta D. (2020). The controlling nutritional status score predicts outcomes of cardiovascular events in patients with heart failure with preserved ejection fraction.. Int. J. Cardiol. Heart Vasc..

[16] Bolat I., Biteker M. (2020). Modified glasgow prognostic score is a novel predictor of clinical outcome in heart failure with preserved ejection fraction.. Scand. Cardiovasc. J..

[17] Adabag S., Rector T.S., Anand I.S. (2014). A prediction model for sudden cardiac death in patients with heart failure and preserved ejection fraction.. Eur. J. Heart Fail..

[18] Adabag S., Langsetmo L. (2020). Sudden cardiac death risk prediction in heart failure with preserved ejection fraction.. Heart Rhythm.

[19] Ghafoor A., Pedersen R., Zlochiver V. (2020). Novel risk stratification score for HFpEF and AFIB: HAD-AFIB.. J. Card. Fail..

[20] Pugliese N.R., De Biase N., Gargani L. (2021). Predicting the transition to and progression of heart failure with preserved ejection fraction: A weighted risk score using bio-humoural, cardiopulmonary, and echocardiographic stress testing.. Eur. J. Prev. Cardiol..

[21] Huttin O., Fraser A.G., Lund L.H. (2021). Risk stratification with echocardiographic biomarkers in heart failure with preserved ejection fraction: The media echo score.. ESC Heart Fail..

[22] Pieske B., Tschöpe C., de Boer R.A. (2019). How to diagnose heart failure with preserved ejection fraction: The HFA-PEFF diagnostic algorithm: A consensus recommendation from the Heart Failure Association (HFA) of the European Society of Cardiology (ESC).. Eur. Heart J..

[23] Sun Y., Si J., Li J. (2021). Predictive value of HFA-PEFF score in patients with heart failure with preserved ejection fraction.. Front. Cardiovasc. Med..

[24] Egashira K., Sueta D., Komorita T. (2022). HFA-PEFF scores: Prognostic value in heart failure with preserved left ventricular ejection fraction.. Korean J. Intern. Med. (Korean. Assoc. Intern. Med.).

[25] Zamfirescu M.B., Ghilencea L.N., Popescu M.R. (2021). A practical risk score for prediction of early readmission after a first episode of acute heart failure with preserved ejection fraction.. Diagnostics (Basel).

[26] Garg P., Lewis R.A., Johns C.S. (2021). Cardiovascular magnetic resonance predicts all-cause mortality in pulmonary hypertension associated with heart failure with preserved ejection fraction.. Int. J. Cardiovasc. Imaging.

[27] Zhu W, Cao Y, Ye M (2023). Essen stroke risk score predicts clinical outcomes in heart failure patients with preserved ejection fraction: Evidence from the TOPCAT trial.. Thromb Haemost.

[28] Liu M., Lee A.P., Sun J.P. (2012). Risk stratification for 1 year mortality in patients with heart failure and normal ejection fraction.. Eur. Heart J..

[29] Shen L., Jhund P.S., Anand I.S. (2021). Developing and validating models to predict sudden death and pump failure death in patients with heart failure and preserved ejection fraction.. Clin. Res. Cardiol..

[30] Pocock S.J., Ferreira J.P., Packer M. (2022). Biomarker‐driven prognostic models in chronic heart failure with preserved ejection fraction: The EMPEROR-preserved trial.. Eur. J. Heart Fail..

[31] Pandey A., Omar W., Ayers C. (2018). Sex and race differences in lifetime risk of heart failure with preserved ejection fraction and heart failure with reduced ejection fraction.. Circulation.

[32] Pandey A., LaMonte M., Klein L. (2017). Relationship between physical activity, body mass index, and risk of heart failure.. J. Am. Coll. Cardiol..

[33] Zhu W., Liang W., Ye Z. (2021). Association of physical activity and risk of atrial fibrillation in heart failure with preserved ejection fraction.. Nutr. Metab. Cardiovasc. Dis..

[34] Haass M., Kitzman D.W., Anand I.S. (2011). Body mass index and adverse cardiovascular outcomes in heart failure patients with preserved ejection fraction: Results from the Irbesartan in Heart Failure with Preserved Ejection Fraction (I-PRESERVE) trial.. Circ. Heart Fail..

[35] Pandey A., Berry J.D., Drazner M.H., Fang J.C., Tang W.H.W., Grodin J.L. (2018). Body mass index, natriuretic peptides, and risk of adverse outcomes in patients with heart failure and preserved ejection fraction: Analysis from the TOPCAT trial.. J. Am. Heart Assoc..

[36] Huang P., Guo Z., Liang W. (2021). Weight change and mortality risk in heart failure with preserved ejection fraction.. Front. Cardiovasc. Med..

[37] Chen J., Li M., Hao B. (2021). Waist to height ratio is associated with an increased risk of mortality in Chinese patients with heart failure with preserved ejection fraction.. BMC Cardiovasc. Disord..

[38] Sandesara P.B., Samman-Tahhan A., Topel M., Venkatesh S., O’Neal W.T. (2018). Effect of cigarette smoking on risk for adverse events in patients with heart failure and preserved ejection fraction.. Am. J. Cardiol..

[39] Eggers K.M., Venge P., Lind L. (2013). Mid-regional pro-atrial natriuretic peptide levels in the elderly: Clinical and prognostic implications, and comparison to B-type natriuretic peptides.. Clin. Chim. Acta.

[40] Jensen J., Schou M., Kistorp C. (2020). MR-proANP and incident cardiovascular disease in patients with type 2 diabetes with and without heart failure with preserved ejection fraction.. Cardiovasc. Diabetol..

[41] Putko B.N., Savu A., Kaul P. (2021). Left atrial remodelling, mid-regional pro-atrial natriuretic peptide, and prognosis across a range of ejection fractions in heart failure.. Eur. Heart J. Cardiovasc. Imaging.

[42] Binder C., Poglitsch M., Duca F. (2021). Renin feedback is an independent predictor of outcome in HFpEF.. J. Pers. Med..

[43] Tsai C.H., Pan C.T., Chang Y.Y. (2021). Left ventricular remodeling and dysfunction in primary aldosteronism.. J. Hum. Hypertens..

[44] Ke B., Tan X., Ren L. (2022). Aldosterone dysregulation predicts the risk of mortality and rehospitalization in heart failure with a preserved ejection fraction.. Sci. China Life Sci..

[45] Altay H., Zorlu A., Bilgi M., Erol T., Yilmaz M.B. (2012). Usefulness of parathyroid hormone as a predictor of heart failure with preserved ejection fraction.. Biomarkers.

[46] Fang Z. (2016). Study of sST2 and Cystatin C in the diagnosis and prognosis of patients with heart failure with preserved ejection fraction..

[47] Unsicker K., Spittau B., Krieglstein K. (2013). The multiple facets of the TGF-β family cytokine growth/differentiation factor-15/macrophage inhibitory cytokine-1.. Cytokine Growth Factor Rev..

[48] Wang J., Wei L., Yang X., Zhong J. (2019). Roles of growth differentiation factor 15 in atherosclerosis and coronary artery disease.. J. Am. Heart Assoc..

[49] Izumiya Y., Hanatani S., Kimura Y. (2014). Growth differentiation factor-15 is a useful prognostic marker in patients with heart failure with preserved ejection fraction.. Can. J. Cardiol..

[50] Chan M.M.Y., Santhanakrishnan R., Chong J.P.C. (2016). Growth differentiation factor 15 in heart failure with preserved vs. reduced ejection fraction.. Eur. J. Heart Fail..

[51] Yin D., Yan X., Bai X., Tian A., Gao Y., Li J. (2023). Prognostic value of growth differentiation factors 15 in acute heart failure patients with preserved ejection fraction.. ESC Heart Fail..

[52] Lakhani I., Wong M.V., Hung J.K.F. (2021). Diagnostic and prognostic value of serum C-reactive protein in heart failure with preserved ejection fraction: A systematic review and meta-analysis.. Heart Fail. Rev..

[53] Kanagala P., Arnold J.R., Khan J.N. (2020). Plasma Tenascin-C: A prognostic biomarker in heart failure with preserved ejection fraction.. Biomarkers.

[54] Aramburu-Bodas Ó., García-Casado B., Salamanca-Bautista P. (2015). Relationship between osteoprotegerin and mortality in decompensated heart failure with preserved ejection fraction.. J. Cardiovasc. Med. (Hagerstown).

[55] Luedike P., Alatzides G., Papathanasiou M. (2018). Predictive potential of macrophage migration inhibitory factor (MIF) in patients with heart failure with preserved ejection fraction (HFpEF).. Eur. J. Med. Res..

[56] Roy C., Lejeune S., Slimani A. (2020). Fibroblast growth factor 23: A biomarker of fibrosis and prognosis in heart failure with preserved ejection fraction.. ESC Heart Fail..

[57] Bayes-Genis A., de Antonio M., Vila J. (2014). Head-to-head comparison of 2 myocardial fibrosis biomarkers for long-term heart failure risk stratification: ST2 versus galectin-3.. J. Am. Coll. Cardiol..

[58] Beltrami M., Ruocco G., Dastidar A.G. (2016). Additional value of Galectin-3 to BNP in acute heart failure patients with preserved ejection fraction.. Clin. Chim. Acta.

[59] Vianello E., Dozio E., Tacchini L., Frati L., Corsi Romanelli M.M. (2019). ST2/IL-33 signaling in cardiac fibrosis.. Int. J. Biochem. Cell Biol..

[60] Zhao YN, Li H, Zhao C, Liu GH (2020). ST2 silencing aggravates ventricular remodeling and chronic heart failure in rats by mediating the IL‐33/ST2 axis.. J Tissue Eng Regen Med.

[61] Piper S.E., Sherwood R.A., Amin-Youssef G.F., Shah A.M., McDonagh T.A. (2015). Serial soluble ST2 for the monitoring of pharmacologically optimised chronic stable heart failure.. Int. J. Cardiol..

[62] Hong T., Shaw R.M. (2017). Cardiac T-tubule microanatomy and function.. Physiol. Rev..

[63] Frisk M., Ruud M., Espe E.K.S. (2016). Elevated ventricular wall stress disrupts cardiomyocyte t-tubule structure and calcium homeostasis.. Cardiovasc. Res..

[64] Seidel T., Navankasattusas S., Ahmad A. (2017). Sheet-like remodeling of the transverse tubular system in human heart failure impairs excitation-contraction coupling and functional recovery by mechanical unloading.. Circulation.

[65] Nikolova A.P., Hitzeman T.C., Baum R. (2018). Association of a novel diagnostic biomarker, the plasma cardiac bridging integrator 1 score, with heart failure with preserved ejection fraction and cardiovascular hospitalization.. JAMA Cardiol..

[66] Nuerbahaer R., Peng H. (2018). Research progress of von willebrand factor in cardiovascular diseases.. Xinjiang Med J.

[67] Kleber M.E., Koller L., Goliasch G. (2015). Von Willebrand factor improves risk prediction in addition to N-terminal pro-B-type natriuretic peptide in patients referred to coronary angiography and signs and symptoms of heart failure and preserved ejection fraction.. Circ. Heart Fail..

[68] Harman J.L., Sayers J., Chapman C., Pellet-Many C. (2020). Emerging roles for neuropilin-2 in cardiovascular disease.. Int. J. Mol. Sci..

[69] Matsui Y., Jia N., Okamoto H. (2004). Role of osteopontin in cardiac fibrosis and remodeling in angiotensin II-induced cardiac hypertrophy.. Hypertension.

[70] Sawaki D., Czibik G., Pini M. (2018). Visceral adipose tissue drives cardiac aging through modulation of fibroblast senescence by osteopontin production.. Circulation.

[71] López B., González A., Lindner D. (2013). Osteopontin-mediated myocardial fibrosis in heart failure: A role for lysyl oxidase?. Cardiovasc. Res..

[72] Tromp J., Khan M.A.F., Klip I.J.T. (2017). Biomarker profiles in heart failure patients with preserved and reduced ejection fraction.. J. Am. Heart Assoc..

[73] Watanabe Y., Tatsuno I. (2021). Omega-3 polyunsaturated fatty acids focusing on eicosapentaenoic acid and docosahexaenoic acid in the prevention of cardiovascular diseases: A review of the state-of-the-art.. Expert Rev. Clin. Pharmacol..

[74] Kaikkonen J.E., Jula A., Viikari J.S.A. (2021). Associations of serum fatty acid proportions with obesity, insulin resistance, blood pressure, and fatty liver: The cardiovascular risk in young Finns study.. J. Nutr..

[75] Matsuo N., Miyoshi T., Takaishi A. (2021). High plasma docosahexaenoic acid associated to better prognoses of patients with acute decompensated heart failure with preserved ejection fraction.. Nutrients.

[76] Harada T., Sunaga H., Sorimachi H. (2020). Pathophysiological role of fatty acid‐binding protein 4 in Asian patients with heart failure and preserved ejection fraction.. ESC Heart Fail..

[77] Wu Z.Y., Liu M.J., Tan L.L. (2020). Research progress of tMAO and cardiovascular diseases.. Chinese Journal of Laboratory Diagnosis.

[78] Salzano A., Israr M.Z., Yazaki Y., Heaney L.M., Suzuki T. (2020). Combined use of trimethylamine N-oxide with BNP for risk stratification in heart failure with preserved ejection fraction: Findings from the DIAMONDHFpEF study.. Eur. J. Prev. Cardiol..

[79] Thawabi M., Hawatmeh A., Studyvin S., Habib H., Shamoon F., Cohen M. (2017). Cardiac troponin and outcome in decompensated heart failure with preserved ejection fraction.. Cardiovasc. Diagn. Ther..

[80] Myhre P.L., O’Meara E., Claggett B.L. (2018). Cardiac troponin I and risk of cardiac events in patients with heart failure and preserved ejection fraction.. Circ. Heart Fail..

[81] Llàcer P., Núñez J., Manzano L. (2021). Carbohydrate antigen 125 (CA125) as a prognostic marker in the elderly with acute heart failure and preserved ejection fraction.. Med. Clin..

[82] Nakagawa A., Yasumura Y., Yoshida C. (2021). Prognostic relevance of elevated plasma osmolality on admission in acute decompensated heart failure with preserved ejection fraction: Insights from PURSUIT-HFpEF registry.. BMC Cardiovasc. Disord..

[83] Zhen Z., Liang W., Tan W. (2022). Prognostic significance of blood urea nitrogen/creatinine ratio in chronic HFpEF.. Eur. J. Clin. Invest..

[84] Huang P., Yu Y., Wei F. (2021). Association of long-term SBP with clinical outcomes and quality of life in heart failure with preserved ejection fraction: An analysis of the treatment of preserved cardiac function heart failure with an aldosterone antagonist trial.. J. Hypertens..

[85] Fuchida A., Suzuki S., Motoki H. (2021). Prognostic significance of diastolic blood pressure in patients with heart failure with preserved ejection fraction.. Heart Vessels.

[86] Huang R., Wu R., Lin Y. (2022). Time-averaged cumulative blood pressure and cardiovascular outcomes in heart failure with preserved ejection fraction: Analysis from the treatment of preserved cardiac function heart failure with an aldosterone antagonist trial.. J. Hypertens..

[87] Suzuki K., Claggett B., Minamisawa M. (2021). Pulse pressure, prognosis, and influence of sacubitril/valsartan in heart failure with preserved ejection fraction.. Hypertension.

[88] O’Neal W.T., Sandesara P.B., Samman-Tahhan A., Kelli H.M., Hammadah M., Soliman E.Z. (2017). Heart rate and the risk of adverse outcomes in patients with heart failure with preserved ejection fraction.. Eur. J. Prev. Cardiol..

[89] Shang X., Lu R., Liu M., Xiao S., Dong N. (2017). Heart rate and outcomes in patients with heart failure with preserved ejection fraction.. Medicine (Baltimore).

[90] Oliva F., Sormani P., Contri R. (2018). Heart rate as a prognostic marker and therapeutic target in acute and chronic heart failure.. Int. J. Cardiol..

[91] Mu F. (2015). Relationship between QRS duration and prognosis in elderly patients with acute myocardial infarction.. Zhongguo Laonianxue Zazhi.

[92] Lund L.H., Jurga J., Edner M. (2013). Prevalence, correlates, and prognostic significance of QRS prolongation in heart failure with reduced and preserved ejection fraction.. Eur. Heart J..

[93] Pei J.H., Pu J.L. (2010). The pathogenesis and clinical significance of Tp-Te interval.. Chin J Cardiac Pac Electrophy.

[94] Li G.J., Liu L.M. (2018). The predictive value of increased Tp-Te interval for adverse cardiovascular events in patients with ejection fraction retention heart failure.. Shandong Yiyao.

[95] Shin S.H., Claggett B., Inciardi R.M. (2021). Prognostic value of minimal left atrial volume in heart failure with preserved ejection fraction.. J. Am. Heart Assoc..

[96] Kammerlander A.A., Donà C., Nitsche C. (2020). Feature tracking of global longitudinal strain by using cardiovascular MRI improves risk stratification in heart failure with preserved ejection fraction.. Radiology.

[97] Sakaguchi E., Yamada A., Naruse H. (2023). Long-term prognostic value of changes in left ventricular global longitudinal strain in patients with heart failure with preserved ejection fraction.. Heart Vessels.

[98] Lee S.H., Lhagvasuren P., Seo J. (2022). Prognostic implications of left ventricular global longitudinal strain in patients with surgically treated mitral valve disease and preserved ejection fraction.. Front. Cardiovasc. Med..

[99] Harada T., Yamaguchi M., Omote K. (2022). Cardiac power output is independently and incrementally associated with adverse outcomes in heart failure with preserved ejection fraction.. Circ. Cardiovasc. Imaging.

[100] Harada D., Asanoi H., Noto T., Takagawa J. (2019). Prominent ‘Y’ descent is an ominous sign of a poorer prognosis in heart failure with preserved ejection fraction.. ESC Heart Fail..

[101] Parrinello G., Torres D., Buscemi S. (2019). Right ventricular diameter predicts all-cause mortality in heart failure with preserved ejection fraction.. Intern. Emerg. Med..

[102] Gorter T.M., Hoendermis E.S., van Veldhuisen D.J. (2016). Right ventricular dysfunction in heart failure with preserved ejection fraction: A systematic review and meta-analysis.. Eur. J. Heart Fail..

[103] Zhao Y.H., Zhao L., Yang X.C. (2020). Mechanism of involvement of epicardial adipose tissue in cardiovascular disease.. Int J Cardiovasc Dis.

[104] White I.A. (2016). Cardiac sympathetic denervation in the failing heart: A role for epicardial adipose tissue.. Circ. Res..

[105] Parisi V., Rengo G., Perrone-Filardi P. (2016). Increased epicardial adipose tissue volume correlates with cardiac sympathetic denervation in patients with heart failure.. Circ. Res..

[106] Wu C.K., Tsai H.Y., Su M.Y.M. (2017). Evolutional change in epicardial fat and its correlation with myocardial diffuse fibrosis in heart failure patients.. J. Clin. Lipidol..

[107] Pugliese N.R., Paneni F., Mazzola M. (2021). Impact of epicardial adipose tissue on cardiovascular haemodynamics, metabolic profile, and prognosis in heart failure.. Eur. J. Heart Fail..

[108] Sato Y., Yoshihisa A., Oikawa M. (2019). Hyponatremia at discharge is associated with adverse prognosis in acute heart failure syndromes with preserved ejection fraction: A report from the JASPER registry.. Eur. Heart J. Acute Cardiovasc. Care.

[109] Zhou Y., Fu L., Sun J. (2021). Association between metabolic syndrome and an increased risk of hospitalization for heart failure in population of HFpEF.. Front. Cardiovasc. Med..

[110] Williams D., Stout M.J., Rosenbloom J.I. (2021). Preeclampsia predicts risk of hospitalization for heart failure with preserved ejection fraction.. J. Am. Coll. Cardiol..

[111] Konishi M., Kagiyama N., Kamiya K. (2021). Impact of sarcopenia on prognosis in patients with heart failure with reduced and preserved ejection fraction.. Eur. J. Prev. Cardiol..

